# Discovering Copy Number Variation in Dual-Purpose XinJiang Brown Cattle

**DOI:** 10.3389/fgene.2021.747431

**Published:** 2022-02-11

**Authors:** Jinghang Zhou, Liyuan Liu, Edwardo Reynolds, Xixia Huang, Dorian Garrick, Yuangang Shi

**Affiliations:** ^1^ School of Agriculture, Ningxia University, Yinchuan, China; ^2^ AL Rae Centre for Genetics and Breeding, Massey University, Hamilton, New Zealand; ^3^ College of Animal Science, Xinjiang Agricultural University, Urumqi, China

**Keywords:** copy number variants, dual-purpose cattle, Xinjiang Brown cattle, (SNP), (GBP4)

## Abstract

Copy number variants (CNVs), which are a class of structural variant, can be important in relating genomic variation to phenotype. The primary aims of this study were to discover the common CNV regions (CNVRs) in the dual-purpose XinJiang-Brown cattle population and to detect differences between CNVs inferred using the ARS-UCD 1.2 (ARS) or the UMD 3.1 (UMD) genome assemblies based on the 150K SNP (Single Nucleotide Polymorphisms) Chip. PennCNV and CNVPartition methods were applied to calculate the deviation of the standardized signal intensity of SNPs markers to detect CNV status. Following the discovery of CNVs, we used the R package HandyCNV to generate and visualize CNVRs, compare CNVs and CNVRs between genome assemblies, and identify consensus genes using annotation resources. We identified 38 consensus CNVRs using the ARS assembly with 1.95% whole genome coverage, and 33 consensus CNVRs using the UMD assembly with 1.46% whole genome coverage using PennCNV and CNVPartition. We identified 37 genes that intersected 13 common CNVs (>5% frequency), these included functionally interesting genes such as *GBP4* for which an increased copy number has been negatively associated with cattle stature, and the *BoLA* gene family which has been linked to the immune response and adaption of cattle. The ARS map file of the GGP Bovine 150K Bead Chip maps the genomic position of more SNPs with increased accuracy compared to the UMD map file. Comparison of the CNVRs identified between the two reference assemblies suggests the newly released ARS reference assembly is better for CNV detection. In spite of this, different CNV detection methods can complement each other to generate a larger number of CNVRs than using a single approach and can highlight more genes of interest.

## Introduction

Copy number variants are structural variants caused by insertions, deletions, duplications, and translocations of DNA fragments (Stankiewicz and Lupski, 2010). The length of CNVs range from 50 bp to several Mbp and can be located on all chromosomes (Pirooznia et al., 2015; Lye and Purugganan, 2019). In cattle, the reported genome coverage of CNVs ranges from 1 to more than 10% depending on the detection strategies and the breed of cattle investigated in the study (Prinsen et al., 2016; Letaief et al., 2017; Kommadath et al., 2019). CNVs can intersect genes which can alter their structure and expression, and many investigations on human disease suggest that copy number variation could be the source of pathogenesis (Stankiewicz and Lupski, 2010). Some studies also suggest that CNVs could explain part of the missing heritability problem in genome-wide association studies on some complex traits (Hay et al., 2018; Génin, 2020). CNVs can influence the linkage disequilibrium between SNPs in the same region, and therefore identifying CNVs could help to improve the accuracy of SNP imputation (Couldrey et al., 2017). CNVs can be inherited and can contribute to genomic diversity that could be informative in evaluating the evolution of different animal breeds (Bickhart et al., 2016). The inclusion of CNV information in genomic evaluation models might improve the accuracy of animal evaluation (Hay et al., 2018). Therefore, studying CNVs in a breed could help us to better understand this population.

Xinjiang-Brown (XJ-Brown) cattle are a dairy and beef dual-purpose breed and are a valuable germplasm resource in the field of cattle breeding. The breed is often used for crossbreeding with local cattle breeds and has a large number of hybrid progeny in China (Zhou et al., 2019). While many CNVs have been reported in studies of several different dairy and beef populations (Prinsen et al., 2016; Couldrey et al., 2017; Letaief et al., 2017), there has been limited research on CNVs in XJ-Brown cattle. Exploring the copy number variation in this breed may help reveal the impacts of natural and artificial selection of its long breeding history and could also provide useful reference information on CNVs for closely related cattle breeds. Most CNV studies in cattle populations have used the UMD 3.1 (UMD) reference genome but recently the newer ARS-UCD 1.2 (ARS) version has been released. The ARS reference genome reported higher continuity, accuracy, and completeness compared to the previous assembly (Rosen et al., 2020). However, given some researchers will continue to use the UMD reference genome in their future work, it is still necessary to provide results on both assembly versions. To better understand the genomic variation of XJ-Brown cattle and provide comprehensive CNV results, we conducted CNV detection analyses on both the ARS and the UMD assemblies using PennCNV (Wang et al., 2007a) and CNVPartition (Illumina. GenomeStudio, 2021) methods with 150k SNP genotyping data. We contrasted CNVs, CNV regions (CNVRs), and genes located in CNVRs across genome assemblies and CNV detection methods, and we provide detail on the consensus genes we detected.

## Methods

### Animals, Genotyping and Quality Control of the Custom Genotyping Cluster File

Xinjiang-Brown (XJ-Brown) cattle is a dual-purpose composite breed with ancestral introductions from Kazakh cattle, Brown Swiss cattle, Alatau cattle and Kostroma cattle (Fu et al., 2017). This study included 403 female XJ-Brown cattle as described in a previous GWAS study (Zhou et al., 2019). These animals were sampled from breeding herds which have contributed thousands of bulls to the XJ-Brown population for decades. The study animals were born between 2003 and 2016 with a mean birth year of 2011.

All 403 animals in the study were genotyped on the GGP Bovine 150K SNP BeadChip using the iScan System. SNP genotyping was performed using GenomeStudio (Illumina. GenomeStudio, 2021) based on SNP signal intensity data, the SNP manifest file, and the official standard cluster file. The default reference genome for the GGP Bovine 150k Beadchip provided in the manifest file is UMD. The chip supplier, NeoGen, provided a bpm format map file with ARS coordinates allowing us to make a complete comparison of the results derived from the UMD and ARS genome assemblies.

CNV detection can be impacted by genotyping batch effects. To reduce the batch effect in CNV detection, two custom cluster files of the UMD and ARS versions were trained on the genotypes of XJ-Brown cattle as instructed in an Illumina technical note (Custom, 2017). Initially, three quality control steps were taken: first, we removed individuals with a call rate less than 0.98; second, we removed the SNPs with a call rate less than 0.95; third, we eliminated animals with abnormally large CNV regions on chromosomes. These filters resulted in 386 individuals, and 136,771 SNPs remaining. The cluster file was then trained on autosomes and sex chromosomes separately.

We used the newly trained custom cluster files to call genotypes, and this was followed by another quality control step. We removed samples with a call rate less than 0.95 leaving 397 individuals, and removed SNPs with call rate less than 0.90. This resulted in 137,945 SNPs remaining in the UMD version, and 138,331 SNPs remained in the ARS version. The map, genotype, Log R Ratio and B Allele Frequency information for these remaining individuals and SNP were exported into the Final Report for further analysis.

### Detection of CNVs

PennCNV (Wang et al., 2007a) and CNVPartition (Illumina. GenomeStudio, 2021) methods were used to discover the CNVs in both the UMD and the ARS datasets. The two methods aim to detect CNVs based on the deviation status of the Log R Ratio (LRR) and the B Allele Frequency (BAF). Quality control was performed in GenomeStudio (Illumina. GenomeStudio, 2021) to ensure the two methods used the same dataset.

CNVPartition (Illumina. GenomeStudio, 2021) is a plug-in for GenomeStudio, and requires a trained custom cluster file, as well as signal intensity data and the SNP manifest file (containing map information) to infer CNV. We selected the GC wave adjust function, set the sex chromosome option to false, the minimum probe count to 3 and the confidence threshold to the default value of 35.

PennCNV (Wang et al., 2007a) software requires four input files including a signal intensity file (containing three columns: SNP Name, LRR and BAF), a SNP Map file (containing three columns: SNP Name, Chromosome, Position), a PFB file and a GC-Content file. The PFB file was produced with the compile_pfb.pl function in PennCNV, the GC-Content file was calculated as the percentage of GC content both 1 MB upstream and downstream of the SNP locus in the bovine reference genome (UCSC Genome Browser Downloads, 2013). Additional PennCNV arguments included the “-lastchr” 29 and “-confidence” flags. After the results of the first run, the -fraction 0.2 and -bp flags were used to combine the adjacent CNVs that had a gap size less than 20% of the total length of the CNVs, then the samples with a standard deviation of LRR larger than 0.30, a BAF drift greater than 0.005 and a wave factor larger than 0.1 or smaller than -0.1 were removed via filter_cnv.pl functions.

### Summary of CNVs and Generation of CNV Regions

The R package HandyCNV (Zhou et al., 2021) was used to summarize CNVs and generate CNV regions (CNVRs). The *cnv_clean()* function was used to convert the CNV results into a standard format and make basic summaries. The argument “drop_length = 5” was used to discard the CNVs larger than 5 Mb. The *cnv_summarise_plot()* function was used to make the CNV distribution, frequency and length group plots. The *call_cnvr()* function was used to generate CNVRs for all results, this method merges CNVs that overlap by at least one bp into a CNVR and reports the frequency of each CNVR. Then, the CNVR distribution map was created using the *cnvr_plot()* function. A gain CNVR indicates the region only contains duplicated CNVs which comprise more than two copies, a loss CNVR indicates the region only contains deleted CNVs of zero copies or one copy, and a mixed CNVR indicates that the region contains at least one duplicated CNV and at least one deleted CNV.

### Comparison of CNVs, CNVRs, and Consensus CNVRs

Our analyses produced four final CNV results, we aimed to make comparisons and find the consensus CNVRs among them. The functions *compare_cnv()* and *compare_cnvr()* in HandyCNV (Zhou et al., 2021) were used to make detailed reports. These reports include the comparison of results on both the individual and the population levels as well as comprehensive comparison plots. Consensus CNVRs in this study were defined as CNVRs that passed the common frequency threshold (Sample size ≥ 20) in the union sets of CNVRs. The union sets of CNVRs were generated with the *call_cnvr()* function by combining the CNVs identified using PennCNV and CNVPartition together on the same reference genome. Then, the consensus CNVRs were identified as overlapping regions in the final CNVR distribution map by assigning that consensus list to the *overlap_cnvr* argument in the *cnvr_plot()* function.

### Annotation of Genes and Consensus Genes

The *get_refgene()* function was used to annotate genes for CNVs and CNVRs based on formatted reference gene lists of UMD and ARS from the UCSC website (UCSC Genome Browser Downloads, 2013) . The *call_gene()* function was used to annotate genes for each version of CNV results. The gene frequency was obtained during the annotation process by counting the total number of CNVs that were annotated to intersect the gene. The consensus genes were defined as genes that had passed the common threshold (sample size ≥ 20 which was calculated by the lowest sample size of 393 multiplied by 0.05). Then, the *compare_gene()* function was used to produce a comparison plot for the consensus genes.

### Visualization of Interesting CNVRs

CNV identification methods detect a deviation of BAF and LRR to distinguish different types of copy number, visualization of CNV via the BAF and LRR plots are a direct way to check and validate the CNVs. The functions *cnv_visual()* and *plot_cnvr_panorama()* were used to visualize CNVs and high frequency CNVRs. To explore the results, we used these functions to customize the CNV plot by chromosome, sample, region of interest and target gene.

### Comparison with Known Cattle CNV Databases and Results from the Literature

To compare these results to known cattle CNV databases, a total of nine studies on CNVs of other cattle breeds were downloaded (all of which are UMD reference genome results). Among them, eight CNV results were downloaded from DGVa database (Database of Genomic Varia, 2019) and one CNVR result was extracted from the literature (RaphaëllePrinsen, 2017). The DGVa database (Database of Genomic Varia, 2019) contains CNV results from dozens of cattle breeds. Here, the strategy from Butty et al. (2020) was adopted to process these data, which combined all of the CNV results in DGVa database (Database of Genomic Varia, 2019) to generate a large CNVR list and then compared this list with our results. Xinjiang Brown cattle are closely related to Brown Swiss cattle, therefore, the results of a Brown Swiss cattle population (RaphaëllePrinsen, 2017) were selected for comparison. In addition, the quality of the reference genome assembly may lead to false positive results in the detection of CNVs. Zhou et al. (2016a) reported nine false positive results of CNVR caused by assembly error in UMD reference genome, and these regions were investigated in this study.

## Results

### Differences in Number of Single-Nucleotide Polymorphism Between Assemblies

**FIGURE 1:**
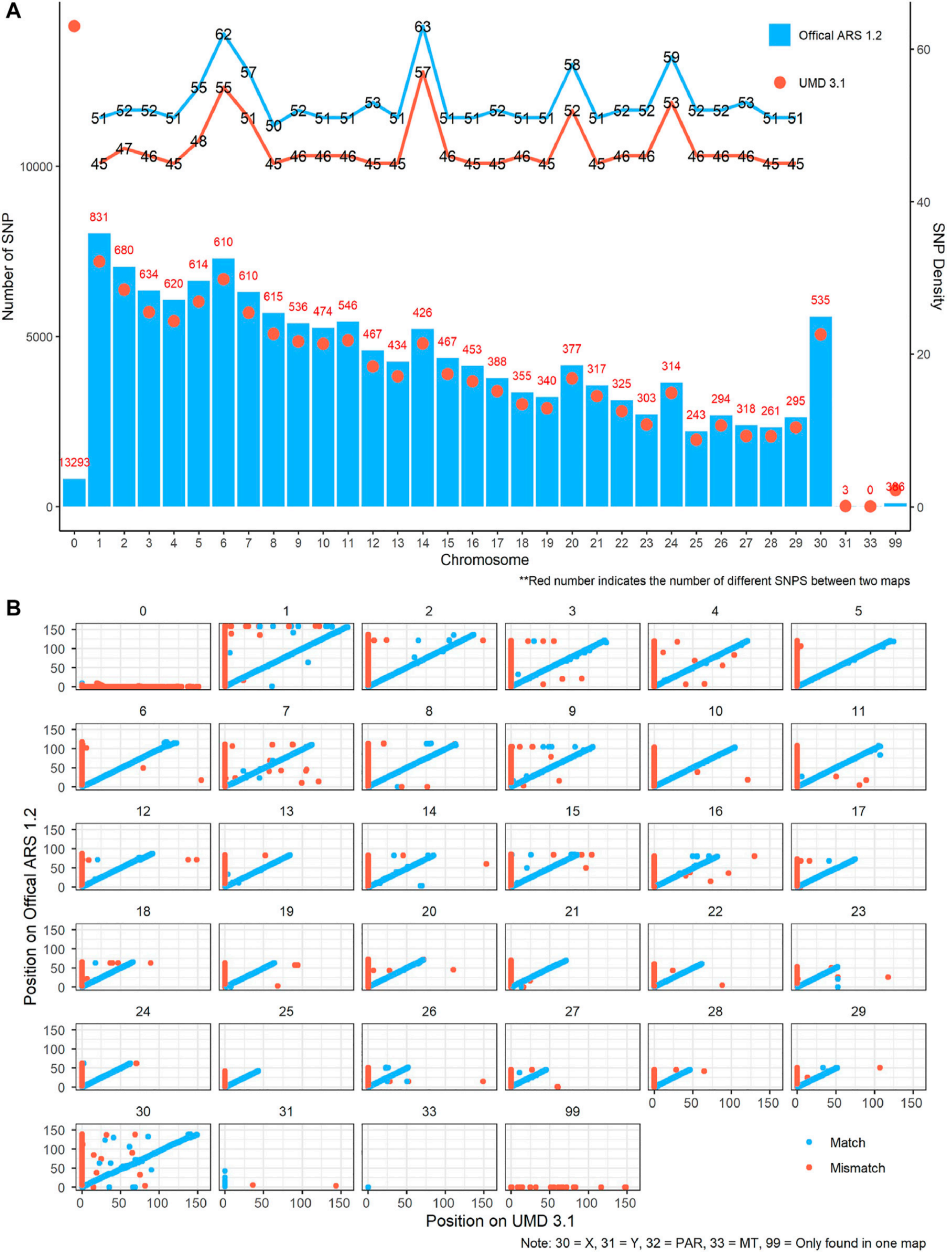
Comparison of single-nucleotide polymorphism (SNP) map between the UMD 3.1 and ARS-UCD 1.2 version. **(A)** SNP number and density difference between the two maps on each chromosome. Blue and red lines indicate the SNP density of the ARS and UMD maps, respectively. Blue bars and red dots represent the quantity of SNP in Athe RS and UMD maps, respectively. **(B)** SNP position difference between the two maps on each chromosome. Blue points indicate the SNP matching on same chromosomes in the two maps, while the red points do the opposite. The points not on the diagonal line indicate that the order of SNPs in the two maps differ greatly on the chromosome.

After quality control, 137,945 SNPs and 138,331 SNPs remained in UMD and ARS maps, respectively. In comparing the two maps, there were 122,963 SNPs (89.1%) located on the same chromosome, 264 SNPs (0.2%) had an unknown position in both UMD and ARS maps, 13,769 SNPs (10.0%) had an unknown position in the UMD map, and 545 SNPs (0.4%) had an unknown position in the ARS map (Table 1). The number of SNPs available for CNV detection on each autosome in the ARS version was larger than that in UMD version, the SNP density ranged from 50–63 SNPs/Mb to 45–57 SNPs/Mb, respectively (Figure 1A). Another difference of the SNPs between the two assemblies were their physical locations. For example, while most SNPs were consistent in both assemblies, some SNPs were on different chromosomes, and some were on the same chromosome but in a completely different order (Figure 1B).

**TABLE 1:** Summary of the difference of single-nucleotide polymorphism (SNP) number between UMD 3.1 and ARS 1.2 assemblies.

Type	Number of SNPs	% In UMD map	% In ARS map
Same chromosome	122,963	89.1	88.9
Both unknown chromosomes	264	0.2	0.2
Different chromosomes	297	0.2	0.2
Unknown chromosome in UMD map	13,769	10.0	10.0
Unknown chromosome in ARS map	545	0.4	0.4
Not found in UMD map	493		0.4
Not found in ARS map	107	0.1	

### Copy Number Variant Statistics From PennCNV and CNVPartition

**FIGURE 2:**
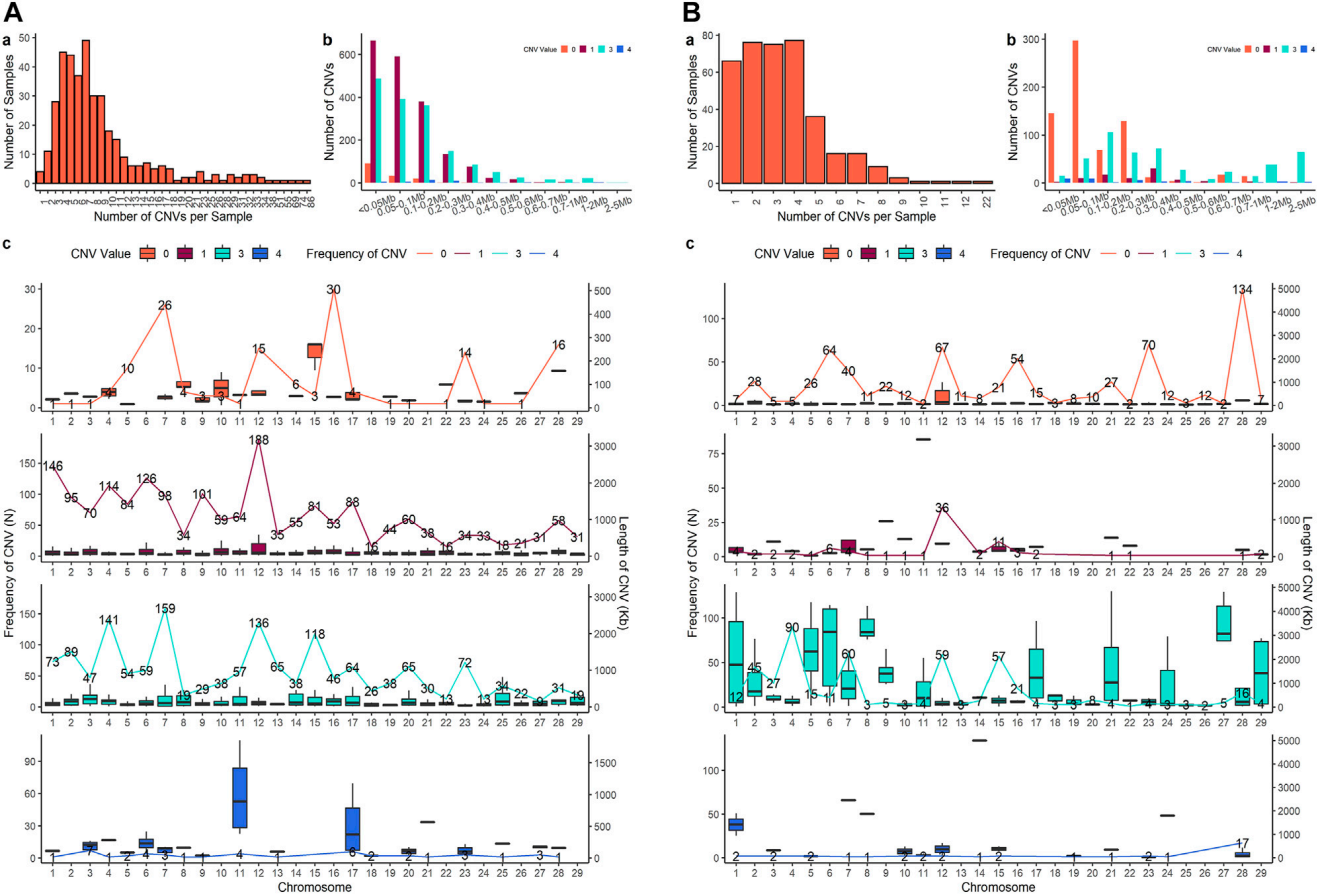
(CNVs) summary plot. **(A)** The results of the Penn-ARS version. **(B)** The results of the Part-ARS version. In each panel, a shows the total number of CNVs in each individual that were detected; b shows the frequency of copies of CNVs in different length groups; c shows CNVs states and distribution on chromosomes. The lines indicate the quantity of CNVs, bar plot presents the length distribution.

**FIGURE 3:**
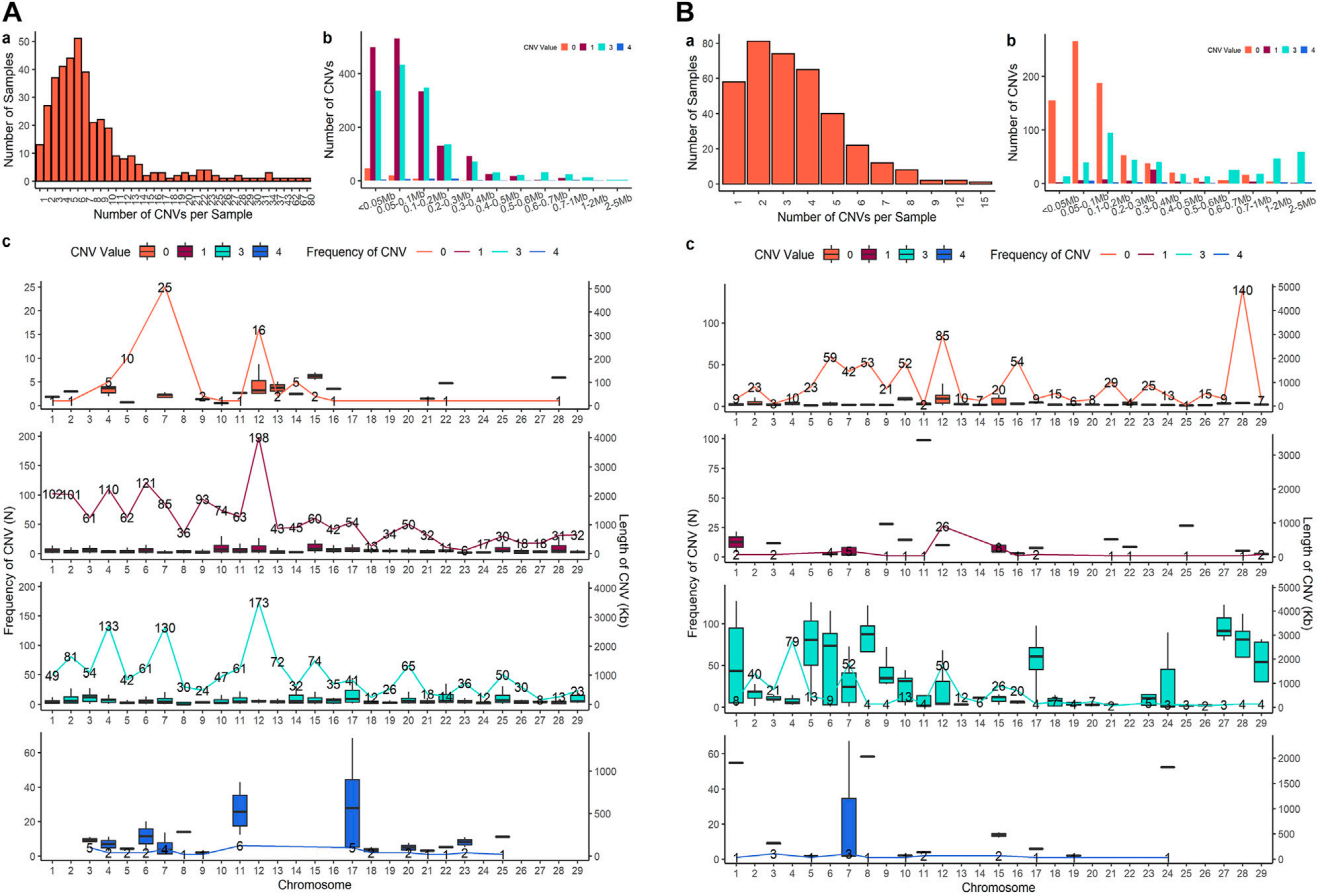
CNVs summary plot. **(A)** The results of the Penn-UMD version. **(B)** The results of the Part-UMD version. In each panel, a shows the total number of CNVs in each individual that were detected; b shows the frequency of copies of CNVs in different length groups; c shows CNVs states and distribution on chromosomes. The lines indicate the quantity of CNVs, bar plot presents the length distribution.

Four sets of CNV results were produced in further analysis. After quality control, 3,686 CNVs were identified from PennCNV with the ARS map (Penn-ARS), 1,293 CNVs from CNVPartition with the ARS map (Part-ARS), 3,200 CNVs from PennCNV with the UMD map (Penn-UMD), and 1,239 CNVs from CNVPartition with the UMD map (Part-UMD). The results from using an ARS map detected more CNVs than using the UMD map. The average number of CNVs per individual were 9.50, 3.42, 8.27, and 3.39 (Table 2), and the largest number of CNVs per individual were 86, 22, 80, and 15 in Penn-ARS (Figure 2A), Part-ARS (Figure 2B), Penn-UMD (Figure 3A), and Part-UMD (Figure 3B) results, respectively.

**TABLE 2:** Statistical description of copy number variant (CNV).

Version	Number of individuals	CNVs per individual	Type	CNV value	Number of CNVs	Average length	Min length	Max length
Penn-ARS	388	9.50	Deletion	0	147	65,640	9,932	343,728
				1	1,891	105,691	1,181	968,527
			Duplication	3	1,604	159,282	5,381	3,181,506
				4	44	285,225	17,406	1,846,515
				In total	3,686	615,839	33,900	6,340,276
Part-ARS	378	3.42	Deletion	0	688	137,420	4,070	986,737
				1	84	327,775	14,229	3,184,065
			Duplication	3	482	786,912	20,505	4,883,451
				4	39	510,859	32,823	4,994,072
				In total	1,293			
Penn-UMD	387	8.27	Deletion	0	75	57,264	9,932	343,900
				1	1,642	118,284	107	917,761
			Duplication	3	1,446	165,302	107	4,018,040
				4	37	267,263	17,392	1,385,828
				In total	3,200			
Part-UMD	365	3.39	Deletion	0	754	148,699	4,510	1,204,452
				1	59	375,120	14,229	3,440,849
			Duplication	3	409	840,778	4,908	4,867,751
				4	17	634,036	58,524	2,336,934
				In total	1,239			

Note. Unit of CNV, length is bp.

All methods detected more deletions than duplications, and all methods identified more three-copy CNVs than four-copy CNVs. PennCNV detected more one-copy than zero-copy deletions whereas the CNVPartition detected more zero-copy than one-copy deletions (Table 2). PennCNV detected considerably more CNVs than CNVPartition, but the average length of each CNV type identified by PennCNV was much smaller than those identified by CNVPartition. We observed the number of one- and three- copy CNVs are much higher than zero- and four-copy CNVs in the PennCNV results and the number of zero- and three- copy CNVs are higher than one- and four- copy CNVs in the CNVPartition results (Figure 2 and Figure 3).

## Copy Number Variant Region Results

There were 931, 279, 821 and 246 CNVRs with total lengths of 150.1 Mb (about 5.5% of the genome coverage), 211.3 Mb (about 7.8% of the genome coverage), 135.0 Mb (about 5.0% of the genome coverage), and 199.2 Mb (about 7.3% of the genome coverage) from the Penn-ARS, Part-ARS, Penn-UMD, and Part-UMD results, respectively (Table 3). There were fewer mixed CNVRs compared with duplication and deletion CNVRs across all CNVR results, but on average, mixed CNVRs had longer length. The ranges of CNVR sizes are from 3.4 kb to 3.4 Mb, 4.0 kb to 4.8 Mb, 3.4 kb to 4.0 Mb, and 4.5 kb to 4.8 Mb for the Penn-ARS, Part-ARS, Penn-UMD, and Part-UMD results, respectively.

**TABLE 3:** Statistical description of copy number variant region (CNVR).

Version	Type	Number	Average length	Min length	Max length	Total length	Genome coverage (%)
Penn-ARS	Gain	307	106,284	7,206	2,426,132	32,629,256	1.2
	Loss	463	121,986	3,431	968,527	56,479,353	2.1
	Mixed	161	378,640	15,108	3,420,406	60,960,964	2.2
	**In total**	**931**				**150,069,573**	**5.5**
Part-ARS	Gain	96	1,310,063	20,505	5,080,301	125,766,089	4.6
	Loss	147	99,070	4,070	968,527	14,563,277	0.5
	Mixed	36	1,971,563	44,203	4,835,353	70,976,269	2.6
	**In total**	**279**				**211,305,635**	**7.8**
Combined ARS	Gain	287	161,168	7,206	3,138,636	46,255,265	1.7
	Loss	480	113,190	4,070	968,527	54,331,035	2.0
	Mixed	187	1,131,005	20,469	5,080,301	211,497,977	7.9
	**In total**	**954**				**312,084,277**	**11.7**
Consensus CNVRs ARS	Gain	4	148,824	229,172	46,380	595,294	0.02
	Loss	4	80,857	159,698	26,551	323,428	0.01
	Mixed	30	1,730,655	4,835,353	100,561	51,919,642	1.91
	**In total**	**38**				**52,838,364**	**1.95**
Penn-UMD	Gain	279	103,115	4,908	762,447	28,769,176	1.1
	Loss	404	123,590	3,435	831,734	49,930,465	1.8
	Mixed	138	407,952	107	4,018,040	56,297,398	2.1
	**In Total**	**821**				**134,997,039**	**5.0**
Part-UMD	Gain	83	1,294,741	22,216	4,867,751	107,463,480	4.0
	Loss	128	138,870	4,510	1,300,277	17,775,377	0.7
	Mixed	35	2,112,119	76,295	4,751,595	73,924,154	2.7
	**In Total**	**246**				**199,163,011**	**7.3**
Combined UMD	Gain	247	216,945	10,881	4,228,858	53,585,459	2.0
	Loss	424	125,080	4,510	1,340,962	53,033,728	2.0
	Mixed	170	1,085,658	107	486,7751	184,561,914	6.9
	**In total**	**841**				**291,181,101**	**10.9**
Consensus CNVRs UMD	Gain	3	165,020	201,664	146,498	495,059	0.02
	Loss	5	67,551	97,170	26,545	337,754	0.01
	Mixed	25	1,526,193	4,451,428	63,835	38,154,832	1.43
	**In total**	**33**				**38,987,645**	**1.46**

**FIGURE 4:**
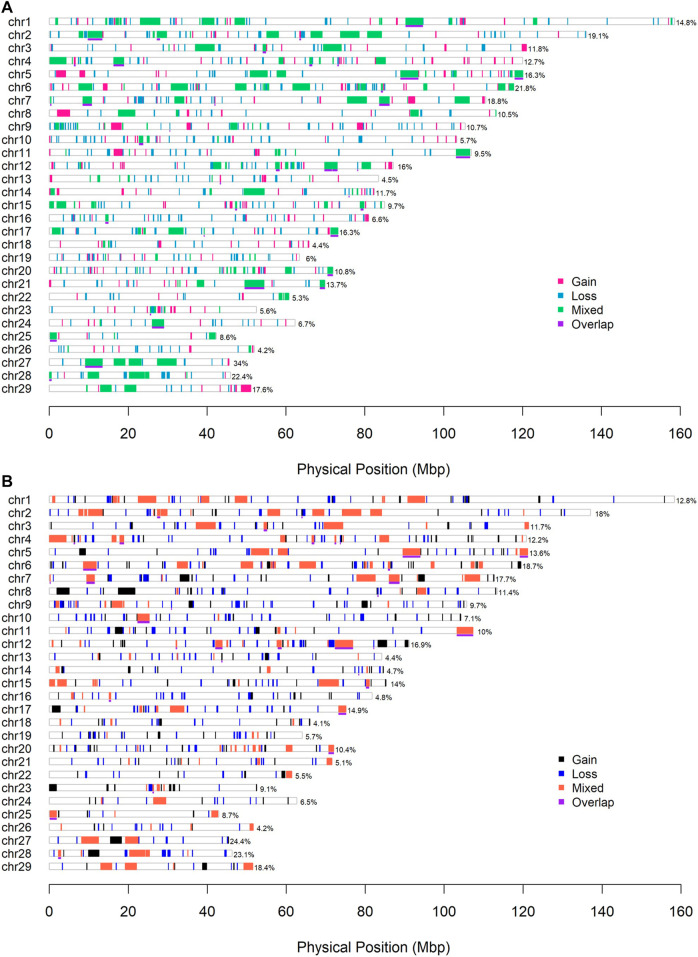
(CNVRs) distribution map. **(A)** The CNVR map with the ARS version. **(B)** The CNVR map with the UMD version. The underlines with purple color indicate the consensus CNVRs between the two detection methods. The percentage number represent the coverage of CNVRs on each chromosome.

Some 954 and 841 CNVRs with a total length of 312.0 Mb (about 11.7% genome coverage) and 291.2 Mb (about 10.9% genome coverage) were in the combined ARS and UMD CNVR lists. The genome coverage of CNVRs on BTA 13, 18 and 26 was less than 5%, the coverage of CNVRs on BTA 27 and 28 was greater than 20% in this sample. There were 38 and 33 CNVRs that passed the consensus frequency threshold (sample size > 20) in the ARS and UMD lists, the total length was 52.84 Mb (about 1.95% genome coverage) and 38.99 Mb (about 1.46% genome coverage), respectively (Table 3). The CNVR distribution map of the complete and the consensus regions in the ARS and UMD maps are presented in Figure 4, respectively.

### Comparison of Copy Number Variants and Copy Number Variant Regions in Different Results

The comparisons were made on both individual and population levels between each pair of CNV results. All results showed that the overlap rate at the population level was higher than that at the individual level.

**FIGURE 5:**
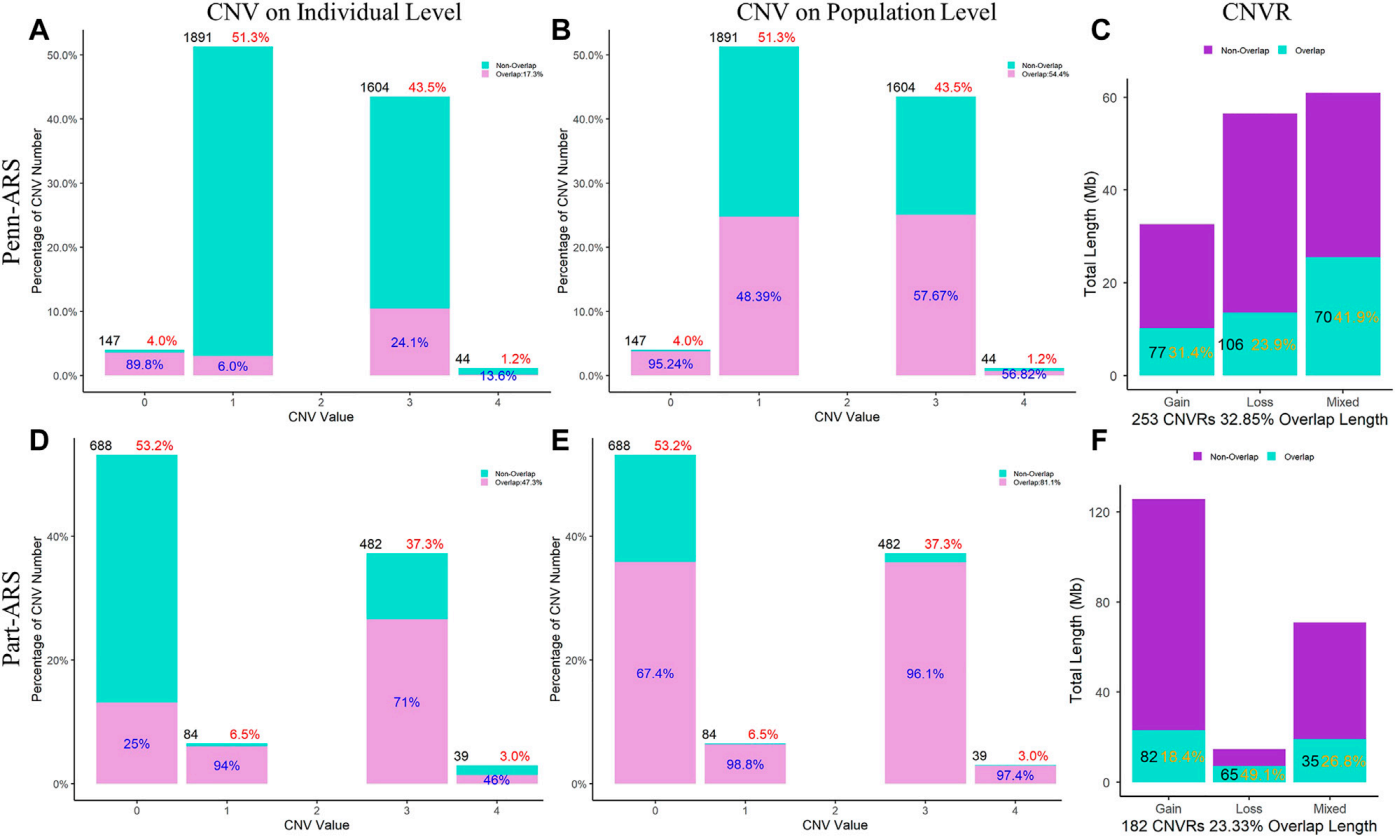
Comparison of CNVs and CNVRs between the Penn-ARS and Part-ARS results. **(A)** and **(D)** are the CNVs comparison results on individual levels. **(B)** and **(E)** are the CNVs comparison results on population level. The percentage in red indicates the proportion of the number of CNVs with different states to the total number of CNVs. The percentage in blue indicates the proportion of the number of overlapping CNVs to each CNV state group. **(C)** and **(F)** are the CNVRs comparison. The percentage in orange represents the proportions of overlapping length of CNVRs to the total length of each type group.

**FIGURE 6:**
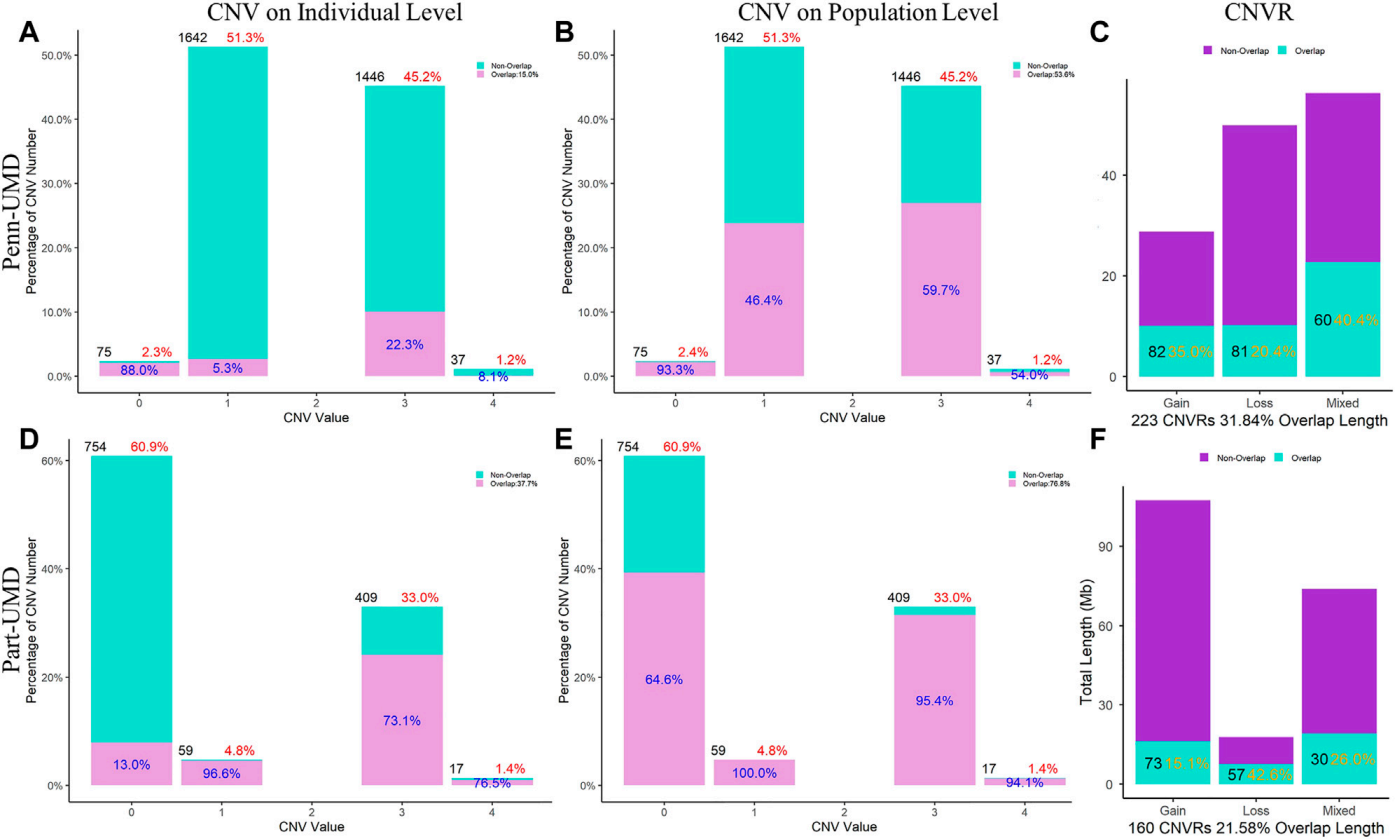
Comparison of CNVs and CNVRs between the Penn-ARS and Part-ARS results. **(A)** and **(D)** are the CNVs comparison results on individual levels. **(B)** and **(E)** are the CNVs comparison results on population level. The percentage in red indicates the proportion of the number of CNVs with different states to the total number of CNVs. The percentage in blue indicates the proportion of the number of overlapping CNVs to each CNV state group. **(C)** and **(F)** are the CNVRs comparison. The percentage in orange represents the proportions of overlapping length of CNVRs to the total length of each type group. Comparison of CNVRs to known CNV datasets.

In the ARS reference genome results, the percentage of overlapped CNVs was 17.3% of Penn-ARS compared to 47.3% of Part-ARS on the individual level (Figures 5A and D), and 54.4% compared to 81.1% on the population level (Figures 5B and E), respectively. In addition, there were 253 CNVRs in Penn-ARS that intersected with 182 CNVRs in Part-ARS, and the total length of overlapped region was 49.29 Mbp corresponding to 32.85% and 23.33% length of the Penn-ARS and Part-ARS results, respectively. We observed similar results when comparing overlapping CNVRs between the Penn-UMD and Part-UMD results (Figure 6). After examining the intersection between the two consensus CNVRs lists, 27 CNVRs were identified that overlapped in 38 and 33 consensus CNVRs in ARS and UMD versions, which comprised about 71.1% and 81.8% of the two consensus CNVRs lists, respectively. There were 44 non-overlapped consensus CNVRs in all results.

Only the results on UMD could be compared with the known datasets, therefore, all positions mentioned in this section are UMD coordinates. A total of 9,934 CNVRs were generated from CNV lists in the DGVa database (Database of Genomic Varia, 2019), with a total length of 745.42 Mbp, involving Holstein, Montbeliard, Normande, Hereford, Charolais, Simmental and other cattle breeds. We identified a total of 565 CNVRs (about 67.2%) in our study were overlapped with 1,369 CNVRs in the DGVa database, and the length of the overlapped regions was 134.45 Mbp, accounting for 46.2% of the total length of XJ-Brown cattle’s CNVRs. We found no intersection between 276 of our CNVR and the DGVa datasets (Database of Genomic Varia, 2019).

We compared our results with those identified in Brown Swiss cattle and found 141 CNVRs in XJ-Brown cattle overlapped with 215 CNVRs in Brown Swiss cattle with a total length of 37.5 Mbp, accounting for 12.9% of the CNVR length in this experiment and 65.6% of Brown Swiss cattle’s CNVR length. Among the 276 CNVRs that had no intersection with DGVa datasets (Database of Genomic Varia, 2019). only nine overlapped with the Brown Swiss cattle’s CNVRs, and the remainder were unique to the XJ-Brown population. In addition, there were three CNVRs (CNVR_70, CNVR_601 and CNVR_609) in our results which overlapped with the false positive regions that were reported by Zhou et al. (2016a), however they were all low-frequency CNVRs.

CNVRs with relatively higher frequency are presented in Table 4, which suggests these higher frequency regions are more likely to be detected by the two methods and are more likely to overlap with other breeds. There is only one CNVR in the higher frequency list in XJ-Brown cattle that has no intersection to both the DGVa (Database of Genomic Varia, 2019) and the Brown Swiss cattle (RaphaëllePrinsen, 2017) datasets. This CNVR was CNVR_146 and was located on BTA4:72297257-72561411 in the Combined-UMD result.

**TABLE 4:** Cross-validation results of CNVR with a sample size greater than 20 in different methods.

Version	Sample ≥ 20	N of overlap	N of non-overlap	Overlap proportion (%)
Penn-ARS vs. Part-ARS	31	28	3	90.3
Part-ARS vs. Penn-ARS	14	14	0	100.0
Penn-UMD vs. Part-UMD	28	27	1	96.4
Part-UMD vs. Penn-UMD	14	13	1	92.9
Combined-UMD vs. Database of Genomic Varia (DGVa)	33	32	1	97.0
Combined-UMD vs. (Brown Swiss) BS	33	29	4	87.9

Note. The numbers in the table correspond to the result of the name with bold type in the version column.

### An Example Showing the Difference of Single-Nucleotide Polymorphisms in a The Same Consensus Copy Number Variant Region

**FIGURE 7:**
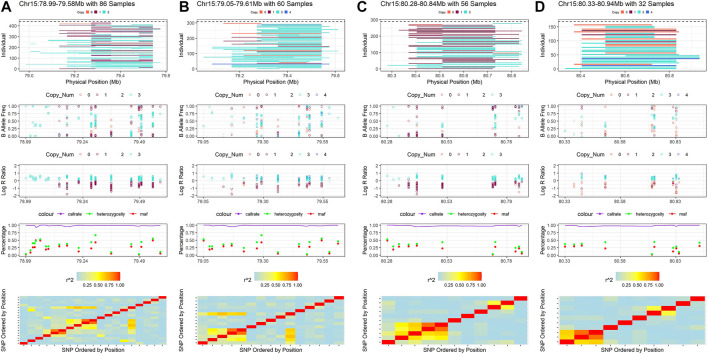
Example of the consensus CNVR that detected by all inference strategies. **(A)** is the visualization result of CNVR of Penn-ARS. **(B)** is the visualization result of CNVR of Part-ARS. **(C)** is the visualization result of CNVR of Penn-UMD. **(D)** is the visualization result of CNVR of Part-UMD.

Some CNVRs could be cross-validated between the four detection methods, however, we observed that the samples in which CNVs are detected may differ between CNV detection methods and between genome assemblies. The differences between reference genomes may be due to the different number and order of SNPs that were used to detect the CNVRs. We present a CNVR on BTA 15 as an example to illustrate these differences (Supplementary Table S1; Figure 7). This CNVR was identified by all methods and a total of 22 unique SNPs in the two maps were found in the four results. However, the number of SNP, start SNP and end SNP were slightly different in each result. In this example, Penn-ARS (CNVR_622, BTA 15:78.99–79.58 Mb) consisted of 19 SNPs, Penn-UMD (CNVR_561, BTA 15:80.28–80.84 Mb) consisted of 15 SNPs, Part-ARS (CNVR_180, BTA 15:79.05–79.61 Mb) consisted of 11 SNPs, and Part-UMD(CNVR_156, BTA 15:80.33–80.94 Mb) consisted of 10 SNPs (Supplementary Table S1). The differences in CNVR breakpoints were not only due to the different reference genomes, the order and location of SNPs on the assemblies, but also the detection methods that were used. For instance, on the ARS assembly, Penn-ARS has five SNPs more in the start region than Part-ARS, but one SNP less in the end region, this variation demonstrates the differences in the two detection methods applied. As can be seen from the visualization results of these CNVRs (Figure 7), the additional SNPs in the two results were only detected in a few samples; The results on the UMD assembly had similar trends to the ARS results.

The SNP differences between different reference genomes could also be observed through this example. In this CNVR on BTA15, there was one SNP with unknown position in the ARS map but six SNPs in the UMD map. In addition, two SNPs had a different order in the two genome assemblies. When we ignored the missing SNPs in this region, the BovineHD1500023413 was located on the fifth loci in ARS map, but on the ninth loci in the UMD map; Another SNP, BovineHD1500023509 was located on the 11th loci in the ARS map, but on the 80th position in the UMD map (Supplementary Table S1).

In view of the two observations above, the samples detected in each result were also different. In example, a total of 86 samples were detected by Penn-ARS, 60 samples were detected by Part-ARS, 56 samples were detected by Penn-UMD and only 32 samples were detected by Part-UMD. When Penn-ARS and Part-ARS results were combined, a total of 106 non-duplicated samples were found, and when Penn-UMD and Part-UMD results were combined, a total of 63 independent samples were found. When all the results are combined, a total of 120 unique samples were obtained (Supplementary Table S1). It shows that different methods can be verified and complementary to each other, and the results combined with multiple strategies can provide more selection samples for the subsequent experimental design.

### Consensus Genes

**FIGURE 8:**
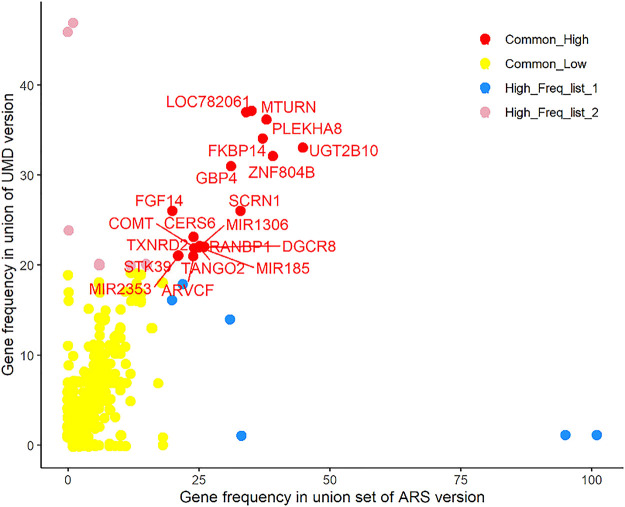
Consensus genes among different results. There are 20 genes in red color that are shown at high frequency in both the ARS and UMD results.

The ARS union set (a combined set of Penn-ARS and Part-ARS results) had 1428 genes annotated in 463 CNVRs, and among these, 277 genes were annotated in 24 consensus CNVRs. After filtering these genes by frequency (>5%), a total of 26 genes (about 9.4%) remained in the ARS union set (Supplementary Table 2). A total of 1,370 genes were annotated in 417 CNVRs in the UMD union-set. Of these genes, 272 were annotated in 18 consensus CNVRs (Supplementary Table 3). After filtering the genes by their frequency, only 31 genes (about 11.4%) remained in the UMD union set. Combining results between assemblies resulted in 37 unique genes, of which 20 genes had common frequency in both the ARS and UMD results (Figure 8), six genes only had common frequency in ARS, and the remaining 11 genes only had common frequency in UMD results. All 37 consensus genes were in 11 and 12 CNVRs in the combined ARS and UMD results, respectively. The most frequent CNVR was CNVR_845 (BTA 23: 25,560,755–25,730,370) and was identified in the ARS analyses, it intersected the *BLA-DQB*, *LOC100848815* and *BOLA-DRB3* genes.

## Discussion

### The Difference of SNP between the UMD and ARS Maps of Bovine GGP 150K Beadchip and Its Effect on CNV Detection

SNP intensity based CNV detection methods are heavily influenced by SNP density and the order of the SNPs on each chromosome. In this study, two main differences were observed between SNPs on the UMD and ARS maps that affected CNV detection on the Bovine GGP 150k Beadchip. The first difference is the total number of SNPs mapped to chromosomes. Approximately 10% of the SNPs in the UMD SNP manifest file had no physical position on the UMD map and were discarded before CNV detection. Many of these missing SNPs did have a physical position on the ARS SNP manifest file resulting in 13,769 additional SNPs available for CNV detection. This difference in SNP density between reference genomes likely influenced the differences we observed in CNV detection on the same CNV regions. This demonstrates the importance of checking how many SNPs are available before CNV detection, if there are many missing SNPs, then it may be better to try to use a well-assembled map file or an up-to-date reference assembly.

The second difference between genome assemblies is how SNP positions differed between ARS and UMD maps of the SNP chip. The SNP order on chromosomes differed between the UMD and ARS maps, as observed in Figure 1, this is likely due to the relative assembly quality of the reference genomes. CNV detection methods required at least three continuous SNPs with the same status to detect a CNV, therefore, the detection of the CNVs may be interrupted by the order of SNPs on a chromosome (Wang et al., 2007a). Due to the shortage of SNP information in the default UMD manifest file of this Bovine GGP 150K Beadchip, using the ARS assembly when detecting CNVs for this SNP chip is suggested.

In this study, the consensus CNVRs from the two methods for ARS and UMD were reported. While, the ARS reference genome has become the main reference genome in recent studies (Butty et al., 2020; Hu et al., 2020), the official map file distributed for this GGP 150K SNP chip is still the UMD version. As such, reporting CNVRs for XJ-Brown cattle in both versions can provide more complete reference for others who are using the same SNP panel.

### Comparison of CNV and CNVR Results of Different Methods

The two methods PennCNV (Wang et al., 2007a) and CNVPartition (Illumina. GenomeStudio, 2021) were used to detect CNVs in this study. We chose these methods because they are commonly used in CNV research, and many studies have verified that the CNVs detected by them are reliable through qPCR validation (Zhang et al., 2014; Wu et al., 2015; Prinsen et al., 2016; Ma et al., 2017; Upadhyay et al., 2017). While CNVRs with the higher frequency tended to have higher validation success rate (Upadhyay et al., 2017) in both methods, the algorithms of the two methods have their own advantages and disadvantages and thus their results can complement each other.

CNVs can be compared at the individual level or the population level. The comparison between individuals can detect the similarity between different methods, while the comparison at the population level can verify the repeatability of CNVs (Zhou et al., 2021). CNVR is the union set of CNVs in all samples, which represents of the entire population. Therefore, comparing CNVR usually refers to differences on the population-level. When comparing CNVs or CNVRs between detection methods, the proportion of overlapped CNVs and CNVRs can be used to compare results more clearly. On the same reference genome, the two methods showed lower consistency at the individual level, such that only 15%–17% of CNVs detected using PennCNV overlapped with 37%–47% of CNVs detected using CNVPartition. The proportion of overlapped CNV numbers at population level was much higher, such that 53%–54% of PennCNV results overlapped with 76%–81% of CNVPartition results. The proportion of CNVR results overlapped by the two methods was also relatively low at 23%–33%. Overall, the consistency of the two methods is low at the individual level, but the repeatability is high at the population level. In particular, the CNVR regions with high frequency had a large proportion of concordance between the two methods.

### Consensus CNVRs in Xinjiang Brown Cattle and Their Comparison with Known Databases

The consensus CNVRs were defined as the CNVRs in the same reference genome that passed the common frequency threshold (Frequency ≥ 0.05) in the union sets of CNVs from the two detection methods. There are two advantages to get consensus CNVR in this way. First, combining multiple CNV results in the same reference genome together to then generate CNVRs will generate the complementary CNVRs list directly. Second, the common thresholds will exclude some low frequency CNVRs and keep the most reliable results.

The CNV results collected from the DGVa database are on the UMD reference genome. Therefore, this study only used the UMD union set to compare with it. The comparison results of this study showed that CNVRs with a detection rate greater than 5% of the sample size were also observed in either the two detection methods or the previously reported studies. After comparing the results of this study with the DGVa or Brown Swiss datasets, we identified a large number of population-specific CNVR, but we could not confirm that these regions were XJ-Brown specific CNVs. This may be because the reference data cited is limited, or because the reliability of low frequency CNVRs is unknown. Even with a high frequency CNVR, there is no way to confirm whether a region is breed-specific, because accurate genotyping of CNVs cannot be obtained from SNP chip data. However, the SNP chip has low cost advantages so large sample sizes can be obtained, and it can still provide some valuable information to infer copy number variation. According to the results of this study, the higher the frequency of CNVR, the higher the accuracy of the results. Therefore, it is suggested that the regions with higher population frequency should be preferentially selected for subsequent analysis when using the PennCNV or CNVPartition methods to detect CNV.

### Consensus Genes between ARS and UMD Versions and Functional Annotation

CNVs can delete or duplicate genes and those changes may impact gene expression and protein function (Fukunaga et al., 2020), therefore identifying these genes is an important part of CNV studies. Here the consensus gene was defined as an annotated gene that intersected at least one CNVR in our results and passes the frequency threshold (occurring in >5% of samples). This approach has two advantages. First, it can filter out the lower frequency genes that may cause false positive results. Because the breakpoints of CNVs of samples are typically not identical in a CNVR, some genes may have low frequency even when located in a high frequency CNVR. As mentioned in the annotation of consensus CNVR results, only 9.4% and 11.4% genes with high frequency CNV status were in the consensus CNVRs. Second, our approach can help to compare the CNVR results between different reference genomes. For example, in this study, the consensus CNVRs were the common CNVRs in two detection methods but they are different in ARS and UMD. This is because some CNVRs failed to convert their coordinates between ARS and UMD results, so that these CNVRs cannot be directly compared between references. We assumed most genes have a unique name and are consistent in different reference genome databases, comparing the frequency of genes by gene names provided an opportunity to make comparison between different CNVR results from the different reference genomes (ARS and UMD). We were more interested in the genes that have structural variants intersecting at higher frequencies.

We identified 37 consensus genes which were predicted to be impacted by a CNVR in at least one of our analyses. Several genes have been reported to intersect CNVs in previous cattle studies, such as the *GBP4* (Guanylate binding protein 4) gene. *GBP4* is an immune related gene, and structural variants intersecting *GBP4* have been reported at common frequencies in multiple cattle populations (Sciences, 2017; Cao et al., 2018; Zhou et al., 2016b). An increased copy number of *GBP4* gene was associated with decreased mRNA expression levels of *GBP4* and *GBP2* genes and associated with significant negative impacts to adult cattle stature (Cao et al., 2018). *BLA-DQB*, *BOLA-DRB3* and *LOC100848815* (Synonyms: *BOLA-DQA1*) genes located at BTA 23: 25,560,755–25,730,370 bp (ARS) are members of bovine leukocyte antigen (*BoLA*) gene families and have been associated with immune response and adaption in cattle studies (Rastislav and Mangesh, 2012; Mei et al., 2020). Several studies have investigated the frequency of CNVs of *BoLA* genes and these have been linked to immune-related phenotypes (Wang et al., 2007b; Fukunaga et al., 2020). The *MAPK8IP3*, *NME3*, *MRPS34*, *SPSB3*, *NUBP2* genes are in a mixed type CNVR located on BTA 25: 208,098–1,769,456 bp (ARS). They have been reported as being related to circulating IGF-binding protein-3 (*IGFBP-3*) concentration in a genome-wide meta-analysis in human studies (Teumer et al., 2016). Structural variation of the *LOC782061* (Synonyms: *AKR1C4*) gene on BTA 13: 43,322,783–43,433,183 (ARS) which is one of the aldo-keto reductase family members and has been shown to relate to expression level during the Bovine estrous cycle (Forde et al., 2011) and in key stages of cattle embryo development (Forde et al., 2009). The consensus CNVR_75 (BTA 2: 27,318,294–27,979,992, ARS version) contains four high frequency genes which are the *NOSTRIN*, *CERS6*, *MIR2353* and *STK39* genes. *CERS6* is involved in lipid and sphingolipid metabolism pathways, and is potentially involved in endocrine control of lactation in dairy cows (McFadden and Rico, 2019). The identification of genes intersected by CNVs at high frequencies in XJ-brown cattle present areas of further research into the potentially functional impacts of the CNV.

## Conclusion

In summary, two CNV detection methods were employed in this study to infer CNVs on each of the ARS-UCD 1.2 and UMD 3.1 genome assemblies. The detailed comparisons of CNVs, CNVRs and annotated genes presented are the most complete CNV results reported in XJ-Brown cattle. A significant proportion of SNP are missing their location in the default UMD 3.1 map file of the GGP Bovine 150k Beadchip, which suggests that improved detection of CNV can be obtained by using the ARS-UCD 1.2 reference genome. A total of 44 common consensus CNVRs were identified with high agreement between reference genomes, and 37 consensus genes were annotated in all CNVRs list, and these results will be helpful for the subsequent experimental design of functional verification of genes impacted by CNVs.

## Data Availability

The CNV lists and entire R script of post-analysis of CNVs in this study can be found at GitHub repositories https://github.com/JH-Zhou/R-Script-of-CNVs-in-XJBrown-Cattle

## Abbreviations

ARS, ARS-UCD 1.2 reference genome; CNV, copy number variant; CNVR, copy number variant region; SNP, single nucleotide polymorphism; UMD, UMD 3.1 reference genome.

## References

[B1] Bickhart D. M. Xu L. Hutchison J. L. Cole J. B. Null D. J. Schroeder S. G. (2016). Diversity and Population-Genetic Properties of Copy Number Variations and Multicopy Genes in Cattle. DNA Res. 23, 253–262. 10.1093/dnares/dsw013 27085184 PMC4909312

[B2] Butty A. M. Chud T. C. S. Miglior F. Schenkel F. S. Kommadath A. Krivushin K. (2020). High Confidence Copy Number Variants Identified in Holstein Dairy Cattle from Whole Genome Sequence and Genotype Array Data. Sci. Rep. 10, 8044. 10.1038/s41598-020-64680-3 32415111 PMC7229195

[B3] Cao X.-K. Huang Y.-Z. Ma Y.-L. Cheng J. Qu Z.-X. Ma Y. (2018). Integrating CNVs into Meta-QTL Identified GBP4 as Positional Candidate for Adult Cattle Stature. Funct. Integr. Genomics 18, 559–567. 10.1007/s10142-018-0613-0 29737453

[B4] Couldrey C. Keehan M. Johnson T. Tiplady K. Winkelman A. Littlejohn M. D. (2017). Detection and Assessment of Copy Number Variation Using PacBio Long-Read and Illumina Sequencing in New Zealand Dairy Cattle. J. Dairy Sci. 100, 5472–5478. 10.3168/jds.2016-12199 28456410

[B5] Custom (2017). Cluster File Creation for Improved Copy Number Analysis. Availableat: www.illumina.com/techniques/microarrays/array-data- .

[B6] Database of Genomic Variants Archive (2019). Database of Genomic Variants Archive. Availableat: https://www.ebi.ac.uk/dgva/data-download .

[B7] Forde N. Beltman M. E. Duffy G. B. Duffy P. Mehta J. P. O'Gaora P. (2011). Changes in the Endometrial Transcriptome during the Bovine Estrous Cycle: Effect of Low Circulating Progesterone and Consequences for Conceptus Elongation. Biol. Reprod. 84, 266–278. 10.1095/biolreprod.110.085910 20881316

[B8] Forde N. Carter F. Fair T. Crowe M. A. Evans A. C. O. Spencer T. E. (2009). Progesterone-Regulated Changes in Endometrial Gene Expression Contribute to Advanced Conceptus Development in Cattle1. Biol. Reprod. 81, 784–794. 10.1095/biolreprod.108.074336 19553605

[B9] Fu X. Lu L. Huang X. Wang Y. Tian K. Xu X. (2017). Estimation of Genetic Parameters for 305 Days Milk Yields and Calving Interval in Xinjiang Brown Cattle. As 08, 46–55. 10.4236/as.2017.81004

[B10] Fukunaga K. Yamashita Y. Yagisawa T. (2020). Copy Number Variations in BOLA‐DQA2 , BOLA‐DQB , and BOLA‐DQA5 Show the Genomic Architecture and Haplotype Frequency of Major Histocompatibility Complex Class II Genes in Holstein Cows. HLA 96, 601–609. 10.1111/tan.14086 33006253

[B11] Génin E. (2020). Missing Heritability of Complex Diseases: Case Solved? Hum. Genet. 139, 103–113. 10.1007/s00439-019-02034-4 31165258

[B12] Hay E. H. A. Utsunomiya Y. T. Xu L. Zhou Y. Neves H. H. R. Carvalheiro R. (2018). Genomic Predictions Combining SNP Markers and Copy Number Variations in Nellore Cattle. BMC Genomics 19. 10.1186/s12864-018-4787-6 PMC5989480 29871610

[B13] Hu Y. Xia H. Li M. Xu C. Ye X. Su R. (2020). Comparative Analyses of Copy Number Variations between *Bos taurus* and *Bos indicus* . BMC Genomics 21, 682. 10.1186/s12864-020-07097-6 33004001 PMC7528262

[B14] Illumina. GenomeStudio.(2021). Illumina. GenomeStudio. Availableat: https://www.illumina.com/techniques/microarrays/array-data-analysis-experimental-design/genomestudio.html .

[B15] Kommadath A. Grant J. R. Krivushin K. Butty A. M. Baes C. F. Carthy T. R. (2019). A Large Interactive Visual Database of Copy Number Variants Discovered in Taurine Cattle. Gigascience 8. 10.1093/gigascience/giz073 PMC6593363 31241156

[B16] Letaief R. Rebours E. Grohs C. Meersseman C. Fritz S. Trouilh L. (2017). Identification of Copy Number Variation in French Dairy and Beef Breeds Using Next-Generation Sequencing. Genet. Sel. Evol. 49. 10.1186/s12711-017-0352-z PMC5655909 29065859

[B17] Lye Z. N. Purugganan M. D. (2019). Copy Number Variation in Domestication. Trends Plant Sci. 24, 352–365. 10.1016/j.tplants.2019.01.003 30745056

[B18] Ma Q. Liu X. Pan J. Ma L. Ma Y. He X. (2017). Genome-wide Detection of Copy Number Variation in Chinese Indigenous Sheep Using an Ovine High-Density 600 K SNP Array. Sci. Rep. 7, 912. 10.1038/s41598-017-00847-9 28424525 PMC5430420

[B19] McFadden J. W. Rico J. E. (2019). Invited Review: Sphingolipid Biology in the Dairy Cow: The Emerging Role of Ceramide. J. Dairy Sci. 102, 7619–7639. 10.3168/jds.2018-16095 31301829

[B20] Mei C. Junjvlieke Z. Raza S. H. A. Wang H. Cheng G. Zhao C. (2020). Copy Number Variation Detection in Chinese Indigenous Cattle by Whole Genome Sequencing. Genomics 112, 831–836. 10.1016/j.ygeno.2019.05.023 31145994

[B21] Pirooznia M. Goes F. S. Zandi P. P. (2015). Whole-genome CNV Analysis: Advances in Computational Approaches. Front. Genet. 06. 10.3389/fgene.2015.00138 PMC4394692 25918519

[B22] Prinsen R. T. M. M. Strillacci M. G. Schiavini F. Santus E. Rossoni A. Maurer V. (2016). A Genome-wide Scan of Copy Number Variants Using High-Density SNPs in Brown Swiss Dairy Cattle. Livestock Sci. 191, 153–160. 10.1016/j.livsci.2016.08.006

[B23] Raphaëlle Prinsen T. M. M. (2017). CNV Detection and Association Studies in the Brown Swiss Cattle Breed. Via Celoria, Italiy: University of Milan.

[B24] Rastislav M. Mangesh B. (2012). BoLA-DRB3 Exon 2 Mutations Associated with Paratuberculosis in Cattle. Vet. J. 192, 517–519. 10.1016/j.tvjl.2011.07.005 21930402

[B25] Rosen B. D. Bickhart D. M. Schnabel R. D. Koren S. Elsik C. G. Tseng E. (2020). De Novo assembly of the Cattle Reference Genome with Single-Molecule Sequencing. Gigascience 9. 10.1093/gigascience/giaa021 PMC7081964 32191811

[B26] Sciences (2017). CNV Detection and Association Studies in the Brown Swiss Cattle Breed.

[B27] Stankiewicz P. Lupski J. R. (2010). Structural Variation in the Human Genome and its Role in Disease. Annu. Rev. Med. 61, 437–455. 10.1146/annurev-med-100708-204735 20059347

[B28] Teumer A. Qi Q. Nethander M. Aschard H. Bandinelli S. Beekman M. (2016). Genomewide Meta‐analysis Identifies Loci Associated with IGF ‐I and IGFBP ‐3 Levels with Impact on Age‐related Traits. Aging Cell 15, 811–824. 10.1111/acel.12490 27329260 PMC5013013

[B29] Ucsc Genome Browser Downloads. (2013). Ucsc Genome Browser Downloads. Available at: https://hgdownload.soe.ucsc.edu/downloads.html#cow. (Accessed August 29, 2021)

[B30] Upadhyay M. da Silva V. H. Megens H. J. Visker M. H. P. W. Ajmone-Marsan P. Bâlteanu V. A. (2017). Distribution and Functionality of Copy Number Variation across European Cattle Populations. Front. Genet. 8, 108. 10.3389/fgene.2017.00108 28878807 PMC5572341

[B31] Wang K. Li M. Hadley D. Liu R. Glessner J. Grant S. F. A. (2007). PennCNV: An Integrated Hidden Markov Model Designed for High-Resolution Copy Number Variation Detection in Whole-Genome SNP Genotyping Data. Genome Res. 17, 1665–1674. 10.1101/gr.6861907 17921354 PMC2045149

[B32] Wang K. Sun D. X. Zhang Y. (2007). Identification of Genetic Variations of Exon 2 of BoLA-DQB Gene in Five Chinese Yellow Cattle Breeds. Int. J. Immunogenet. 34, 115–118. 10.1111/j.1744-313x.2007.00654.x 17373936

[B33] Wu Y. Fan H. Jing S. Xia J. Chen Y. Zhang L. (2015). A Genome-wide Scan for Copy Number Variations Using High-Density Single Nucleotide Polymorphism Array in Simmental Cattle. Anim. Genet. 46, 289–298. 10.1111/age.12288 25917301

[B34] Zhang H. Du Z.-Q. Dong J.-Q. Wang H.-X. Shi H.-Y. Wang N. (2014). Detection of Genome-wide Copy Number Variations in Two Chicken Lines Divergently Selected for Abdominal Fat Content. BMC Genomics 15, 517. 10.1186/1471-2164-15-517 24962627 PMC4092215

[B35] Zhou J. Liu L. Chen C. J. Zhang M. Lu X. Zhang Z. (2019). Genome-wide Association Study of Milk and Reproductive Traits in Dual-Purpose Xinjiang Brown Cattle. BMC Genomics 20. 10.1186/s12864-019-6224-x PMC6842163 31703627

[B36] Zhou J. Liu L. Lopdell T. J. Garrick D. J. Shi Y. (2021). HandyCNV: Standardized Summary, Annotation, Comparison, and Visualization of CNV, CNVR and ROH. CNVR and ROH. 10.1101/2021.04.05.438403 PMC8484803 34603390

[B37] Zhou Y. Utsunomiya Y. T. Xu L. Hay E. H. A. Bickhart D. M. Alexandre P. A. (2016). Genome-wide CNV Analysis Reveals Variants Associated with Growth Traits in *Bos indicus* . BMC Genomics 17, 419. 10.1186/s12864-016-2461-4 27245577 PMC4888316

[B38] Zhou Y. Utsunomiya Y. T. Xu L. Hay E. H. a. Bickhart D. M. Sonstegard T. S. (2016). Comparative Analyses across Cattle Genders and Breeds Reveal the Pitfalls Caused by False Positive and Lineage-Differential Copy Number Variations. Sci. Rep. 6, 29219. 10.1038/srep29219 27381368 PMC4933914

